# An Assessment of Physicians’ Recommendations for Colorectal Cancer Screening and International Guidelines Awareness and Adherence: Results From a Thai National Survey

**DOI:** 10.3389/fmed.2022.847361

**Published:** 2022-04-29

**Authors:** Nonthalee Pausawasdi, Pongkamon Tongpong, Tanawat Geeratragool, Phunchai Charatcharoenwitthaya

**Affiliations:** ^1^Siriraj GI Endoscopy Center, Faculty of Medicine Siriraj Hospital, Mahidol University, Bangkok, Thailand; ^2^Division of Gastroenterology, Department of Medicine, Faculty of Medicine Siriraj Hospital, Mahidol University, Bangkok, Thailand

**Keywords:** colorectal cancer, screening, physician, recommendation, awareness, adherence

## Abstract

**Background:**

Colorectal cancer (CRC) screening uptake is generally low in the Asia Pacific and physicians’ recommendations affect the screening participation.

**Objective:**

The study aimed to assess Thai physicians’ recommendations for CRC screening, and the awareness of and adherence to international guidelines.

**Methods:**

A survey containing questions assessing physicians’ demographic data, screening recommendations, and awareness of the international CRC screening guidelines assessed by clinical vignettes. Independent predictors of physicians’ recommendations for CRC screening were determined by logistic regression analysis.

**Results:**

Five hundred and eighty-sixth of 1,286 (46%) physicians completed the survey, and 58% of them offered CRC screening. The majority of colorectal surgeons (91%) and gastroenterologists (86%) endorsed screening, whereas 35% of primary care physicians recommended screening. The patient’s age was the only factor influencing the physician’s decision to offer CRC screening (OR, 2.75: 95% CI, 1.61–4.67). Colonoscopy was the most recommended modality among specialists, whereas 60% of primary care physicians offered fecal occult blood tests (FOBTs). The guidelines awareness was noted in 81% of participants, with the highest rates among gastroenterologists and colorectal surgeons. Gastroenterologists were more likely to adhere to the guidelines than surgeons, but both recommended shorter interval surveillance colonoscopy than guidelines recommendations in cases of small hyperplastic rectosigmoid polyps.

**Conclusions:**

Recommendations for CRC screening and awareness of guidelines vary among different specialties. A low proportion of primary care physicians recommended screening and colorectal surgeons and gastroenterologists recommended shorter intervals for surveillance of small hyperplastic polyp than suggested by guidelines.

## Introduction

Colorectal cancer (CRC) is the third most prevalent cancer worldwide, with wide geographical variation in incidence and mortality rates (1). CRC screening is recognized as a proven strategy to improve prognosis and survival given the nature of the extended precancerous phase, and the ability to cure precancerous lesions (1–3). The optimal screening approach for each region may differ according to resources, availability of tests and healthcare providers, procedural risks, costs, personal beliefs, and cultural barriers.

Several international guidelines have been published to provide evidence-based recommendations for CRC screening programs. Most guidelines recommend screening for average-risk individuals at 45–50 years of age (4–8). Despite the evident benefits of CRC screening, the participation rate in the Asia Pacific region is generally low, ranging from 1.0 to 49.0% (9–13). In contrast, the United States reported a CRC screening uptake rate of 65.5% (14). A systematic review of screening in the United States showed that facilitator factors (physician recommendation, public education, social network, and self-motivation) and barriers (fear, fatalism, aversion, and cultural barriers) influenced CRC screening uptake rates (15). A multicenter, international study involving 14 countries in the Asia-Pacific area surveyed the population’s attitudes and barriers to CRC screening. The investigators found that physicians’ recommendations and knowledge of screening tests were significant factors in CRC screening uptake (12). The authors stated that promoting physicians’ roles in improving awareness of CRC is essential to implementing a mass screening program to increase screening participation rates.

In Thailand, CRC remains one of the major unsolved national healthcare issues. Colon cancer is the third most frequent cancer in men and the fourth most common cancer in women (16). Furthermore, it is only cancer with increased incidence rates in both sexes (17). The majority of patients with CRC have nodal or distant metastases at their presentations (18, 19). Therefore, a mass screening program is required nationwide to reduce cancer incidence and allow early detection. A national survey study among colorectal surgeons showed that 84% of them offered CRC screening to the average-risk population, using a variety of screening modalities (20). Nonetheless, recommendations from physicians other than colorectal surgeons have not been evaluated.

The primary objective of this study was to evaluate physicians’ recommendations for CRC screening across the country. Also, the present study aimed to determine the proportion of physicians who are aware of the international CRC screening guidelines, and to assess the adherence to guidelines among those with awareness.

## Materials and Methods

### Subjects

A questionnaire-based survey study was conducted. In the context of this study, physicians included resident physicians, primary care physicians, internists, gastroenterologists, and surgeons. The questionnaires were sent to practicing physicians nationwide, including members of the following organizations: (1) the Royal College of Thai Physicians (RCTP); primary care physicians, internists, and resident physicians in internal medicine, (2) the Gastroenterological Association of Thailand (GAT); gastroenterologists, and (3) the Thai Royal College of Surgery (TRCS); general surgeons, colorectal surgeons, and resident physicians in surgery.

### Questionnaire

Content experts in gastroenterology, general surgery, and colorectal surgery were invited to participate in the questionnaire development. The experts reviewed the current CRC screening guidelines, including the 2008 American Gastroenterological Association CRC screening and surveillance guidelines (21). A face-to-face meeting was held to ensure that the questions captured the topic of interest during the questionnaire development. The completed questionnaire was tested with colleagues in both surgical and gastroenterological fields, trainees included, *via* a web-based survey monkey for feedback. A minor revision was made to finalize the questionnaire (Supplementary Material 1). The paper-and-pencil self-administered questionnaires were mailed to participants. In addition, the web-based online survey was done *via* SurveyMonkey.^1^ The two versions of the questionnaire were identical in terms of the questions asked, their wording, and their order of presentation in the survey. The questionnaire contains three domains, including (1) physician demographics, (2) physician’s practice in CRC screening and the awareness of CRC screening guidelines, and (3) physician’s adherence to CRC screening guidelines.

The first domain of the questionnaire assesses the following demographic data and general information: age, sex, area of specialty (general practice, resident physician, internal medicine, general surgery, colorectal surgery, and gastroenterology), years of practice, number of patients seen per week, place of employment (academic center, community hospital, tertiary care center, and private hospital) and geographic location of the workplace (central, north, northeast, east, and south region of the country).

The second domain of the questionnaire was designed to provide insight into physicians’ CRC screening practices and their awareness of guidelines. Participants were asked specific questions about their rate of CRC screening offers, their impression of the appropriate age at which screenings should be initiated and discontinued, factors influencing their decision to recommend CRC screening (age, sex, family history of CRC, comorbidity, reimbursement, and the availability of CRC screening), and questions regarding the modalities of CRC screening [fecal occult blood test (FOBT), including guaiac fecal occult blood test (gFOBT) and immunochemical fecal occult blood test (iFOBT) or fecal immunochemical testing (FIT), barium enema, computed tomography (CT) colonography, flexible sigmoidoscopy, colonoscopy, and blood test for carcinoembryonic antigen (CEA) level].

The last domain of the questionnaire asked gastroenterologists and surgeons participants to rate their own adherence to CRC surveillance guidelines using four clinical vignettes. The following are the four questions that were posed: (1) When do you recommend surveillance in a patient without a family history of CRC who had a normal screening colonoscopy?, (2) When do you recommend surveillance in a patient with a family history of CRC who had normal screening colonoscopy?, (3) When do you recommend surveillance for a patient who had complete polypectomy of villous or tubulovillous adenoma of larger than 1 cm?, and (4) When do you recommend surveillance for a patient who had multiple (<10 polyps) hyperplastic polyps of less than 1 cm at the sigmoid colon and rectum?

### Ethics

The study protocol conformed to the ethical guidelines of the 1975 Helsinki Declaration and has been approved by the Siriraj Institutional Review Board (COA no. Si448/2015). RCTP, GAT, and TRCS did not receive any compensation for their participation in this survey, which was completely voluntary. The questionnaire was anonymous, and no personal information about the respondents was gathered. Participants gave their informed consent to take part in the study.

### Statistical Analysis

Data were summarized using descriptive statistics. Categorical variables were compared using the χ^2^ test. Variables that might influence CRC screening recommendations were identified using logistic regression analysis and summarized with odds ratio (OR) and 95% confidence interval (CI). All statistical testing was performed at the conventional 2-tailed α level of 0.05. SPSS 18.0 software (SPSS, Inc., Chicago, IL, United States) was used to perform all statistical analyses.

## Results

### Demographic Data

**FIGURE 1:**
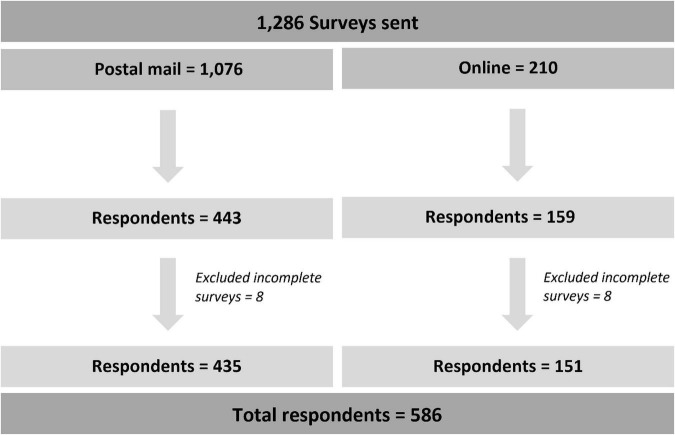
Study flow chart.

The questionnaires were distributed to 1,286 physicians nationwide (postal mail = 1,076 and online = 210). A total of 586 respondents were included in the study indicating a response rate of 46% (Figure 1). The mean age of respondents was 34.0 ± 11.4 years (range: 24–78), and males accounted for 59.7%. Of them, 29.5% were internists, 17.9% were resident physicians in internal medicine, 17.4% were general surgeons, 13.1% were primary care physicians, 13.0% were gastroenterologists, 5.1% were resident physicians in surgery, and 3.9% were colorectal surgeons. Most respondents graduated 6–10 years ago from medical schools (35.2%), 22.0% graduated less than five years ago, and 21.5% graduated more than 20 years ago. One-third of respondents encountered 20–49 patients per week, and 32.6% encountered 50–100 patients per week in the clinic. Thirty percent worked at academic centers and 25.6% in tertiary care hospitals. Regarding workplaces’ geographical location in Thailand, 52.4% lived in the central area, 18.4% in the south, and 15.5% in the north. Characteristics of respondents are shown in Table 1.

**TABLE 1:** Baseline characteristics of respondents.

Baseline characteristics	Responders (*N* = 586)
Age, year	34.0 ± 11.4
Male gender, *N* (%)	350 (59.7)
**Specialty, *N* (%)**	
Internists	173 (29.5)
Resident physicians in internal medicine	105 (17.9)
General surgeons	102 (17.4)
Primary care physicians	77 (13.1)
Gastroenterologists	76 (13.0)
Resident physicians in surgery	30 (5.1)
Colorectal surgeons	23 (3.9)
**Years of practice, *N* (%)**	
≤5 years	129 (22.0)
6–10 years	206 (35.2)
11–15 years	69 (11.8)
16–20 years	56 (9.6)
>20 years	126 (21.5)
**Patients seen per week, *N* (%)**	
≤20	49 (8.4)
21–50	192 (32.8)
51–100	191 (32.6)
>100	154 (26.3)
**Distribution of workplace, *N* (%)**	
Academic centers	176 (30.0)
Tertiary care centers	150 (25.6)
Private hospitals	92 (15.7)
Provincial hospitals	80 (13.7)
Community hospitals	77 (13.1)
Private clinics	11 (1.9)
**Geographic distribution, *N* (%)**	
Central	307 (52.4)
South	108 (18.4)
North	91 (15.5)
Northeast	58 (9.9)
East	22 (3.8)

*Data presented as mean ± standard deviation or number and percentage.*

### Rate of Colorectal Cancer Screening Recommendation

Overall, 58.2% of the respondents offered CRC screening with no variation among different geographic areas and years of practice. Both colorectal surgeons (91.3%) and gastroenterologists (86.8%) were most likely to offer CRC screening, followed by internal medicine residents (65.9%), general surgeons (58.8%), surgical residents (50.4%), and internists (49.7%). In contrast, primary care physicians recommended screening at the lowest rate of 35.1%. Physicians in the community were less likely than specialists to offer screening.

### Factors Influencing Physicians’ Recommendations for Colorectal Cancer Screening

We performed a univariate analysis and determined physicians’ specialty and type of hospital were associated with recommendations for CRC screening. To account for potential confounding factors, physician’s specialty, years of practice, number of patients seen per week, type of hospital, and geographic distribution were included in the multivariate regression model for assessing physician-related factors associated with CRC screening recommendations. We found that colorectal surgeons, gastroenterologists, resident physicians in internal medicine, and general surgeons were more likely to offer screening than primary care physicians. Furthermore, physicians working in academic centers, tertiary care hospitals, and private hospitals were more likely to recommend CRC screening than those working in community hospitals or private clinics (Table 2).

**TABLE 2:** Physician-related factors associated with colorectal cancer screening recommendations.

Factors	Univariate analysis unadjusted odds ratio (95% CI)	*P*-value	Multivariate analysis adjusted odds ratio (95% CI)	*P*-value
**Specialty**		<0.001		<0.001
Primary care physicians	Reference		Reference	
Colorectal surgeons	20.05 (4.30–93.45)		13.18 (2.56–67.86)	
Gastroenterologists	11.28 (4.96–25.65)		7.72 (3.08–19.39)	
Resident physicians	3.14 (1.69–5.56)		2.22 (1.02–4.84)	
General surgeons	2.62 (1.37–5.01)		2.09 (0.97–4.50)	
**Years of practice**		0.056		0.787
<10 years	Reference		Reference	
15–20 years	1.64 (1.07–2.52)		1.09 (0.65–1.86)	
>20 years	1.34 (0.88–2.03)		0.88 (0.50–1.54)	
**Number of patients seen per week**		0.084		0.296
<20 patients/week	Reference		Reference	
21–50 patients/week	1.34 (0.68–2.64)		1.58 (0.77–3.24)	
51–100 patients/week	0.92 (0.47–1.80)		1.03 (0.49–2.15)	
>100 patients/weeks	0.78 (0.39–1.53)		1.26 (0.58–2.45)	
**Type of hospital**		0.008		0.010
Primary/private clinics	Reference		Reference	
Private hospitals	6.67 (3.37–13.18)		4.43 (1.99–9.86)	
Academic centers	3.67 (2.09–6.45)		1.93 (0.92–4.06)	
Tertiary care centers	2.23 (1.27–3.94)		1.80 (0.93–3.46)	
**Geographic distribution**		0.600		0.764
North	Reference		Reference	
Northeast	0.84 (0.43–1.61)		1.34 (0.64–2.79)	
East	1.29 (0.50–3.32)		1.04 (0.37–2.94)	
Center	1.67 (1.04–2.69)		1.36 (0.79–2.33)	
South	0.83 (0.48–1.45)		1.06 (0.57–1.96)	

*Parameters included for multivariate analysis model: physicians’ specialty, years of practice, number of patients seen per week, type of hospital and geographic distribution.*

The patient’s age was the only significant patient factor influencing the physician’s decision to offer CRC screening (OR, 2.75: 95% CI, 1.61–4.67). Physicians’ intention to recommend CRC screening was not affected by other patient-related factors (such as gender, family history of CRC, and comorbidities), reimbursement policies, or hospital facility, as shown in Table 3.

**TABLE 3:** Univariate analysis of patient-related factors associated with colorectal cancer screening recommendations.

Factor influencing the decision to offer CRC screening	Odds Ratio (95% CI)	*P*-value
Age (yes vs. no)	2.75 (1.61–4.67)	<0.001
Gender (yes vs. no)	0.83 (0.54–1.23)	0.407
Family history of CRC (yes vs. no)	1.40 (0.40–4.91)	0.749
Comorbidities (yes vs. no)	0.89 (0.60–1.33)	0.579
Reimbursement policies (yes vs. no)	1.37 (0.84–2.22)	0.209
Hospital facility (yes vs. no)	1.08 (0.75–1.57)	0.654

*CRC, colorectal cancer.*

### Screening Modalities

Almost all respondents’ workplaces (96.9%) had a tool to perform CRC screening. FOBT was the most commonly accessible option for 88.6% of respondents. Colonoscopy was the second most prevalent test, accounting for 81.9%, followed by barium enema (68.4%). Colonoscopy was advised by the majority of respondents (68.9%), while FOBT was offered by 45.1%. Other screening modalities, such as flexible sigmoidoscopy combined with barium enema, flexible sigmoidoscopy alone, CT colonoscopy, and serum CEA, were all recommended at the rates of 5–10%. Colonoscopy was the test of choice recommended by gastroenterologists and colorectal surgeons, whereas primary care physicians preferred FOBT. Colonoscopy was offered as a screening test by 96.1% of gastroenterologists and 91.3% of colorectal surgeons. The screening methods offered by each specialty and the availability of each method in their facility are shown in Table 4.

**TABLE 4:** Colorectal cancer screening method by each specialty.

Factors	Primary care physician (*N* = 77)	Internist (*N* = 173)	Resident physician (*N* = 135)	General Surgeon (*N* = 102)	Colorectal Surgeon (*N* = 23)	Gastroent-erologist (*N* = 76)	*P*-value
**Screening method, *N* (%)**							
FOBT	45 (58.4)	86 (49.7)	57 (42.2)	42 (41.2)	7 (30.4)	27 (35.5)	0.020
CEA	8.4 (10.9)	29 (16.8)	4 (3.0)	17 (16.7)	2 (8.7)	5 (6.6)	0.001
CT colonography	0 (0.0)	8 (4.6)	6 (4.4)	5 (4.9)	5 (21.7)	7 (9.2)	0.003
Sigmoidoscopy and barium enema	12 (1.6)	8 (4.6)	15 (11.1)	26 (25.5)	2 (8.7)	7 (9.2)	<0.001
Sigmoidoscopy	12 (1.6)	9 (5.2)	5 (3.7)	8 (7.8)	1 (4.1)	2 (2.6)	0.420
Colonoscopy	33 (42.9)	100 (57.8)	100 (74.1)	77 (75.5)	21 (91.3)	73 (96.1)	<0.001
**Available method, *N* (%)**							
No screening tool	11 (14.3)	5 (2.9)	0 (0.0)	2 (2.0)	0 (0.0)	0 (0.0)	<0.001
FOBT	59 (76.6)	156 (90.2)	127 (94.1)	86 (84.3)	20 (87.0)	71 (93.4)	0.003
Sigmoidoscopy	16 (20.8)	78 (45.1)	98 (72.6)	61 (59.8)	15 (65.2)	41 (54.0)	<0.001
Barium enema	31 (40.3)	113 (65.3)	105 (77.8)	75 (73.5)	19 (82.6)	58 (76.3)	<0.001
Colonoscopy	39 (50.7)	133 (76.9)	126 (93.3)	87 (85.3)	23 (100.0)	72 (94.7)	<0.001
CT colonoscopy	16 (20.8)	53 (30.6)	70 (51.9)	33 (32.4)	15 (65.2)	45 (59.2)	<0.001

*Data presented as percentage of screening method by each specialty. FOBT, fecal occult blood test; CEA, carcinoembryonic antigen; CT; computed tomography.*

### Adherence to Colorectal Cancer Screening Guideline

Overall awareness of the CRC guidelines was 81.1%, with the highest rates noted among gastroenterologists and colorectal surgeons. Three hundred and twenty-nine (56.1%) of 586 respondents began screening average-risk people at age 50 as recommended by the international guidelines. Approximately 90% of gastroenterologists and colorectal surgeons adhered to the guideline recommendation regarding the start age for screening. The decision to discontinue screening at the age of 80 was noted in 44.0% of respondents. Only 29.0% agreed to cease screening at the age of 75, as recommended by the guidelines. Notably, 77.0% of respondents would continue to offer screening if the government covered the cost.

The clinical vignettes model around the CRC screening consensus guidelines were applied exclusively to gastroenterologists, general surgeons, and colorectal surgeons (Table 5). Gastroenterologists were more likely to adhere to the guidelines than surgeons, but both specialists recommended shorter interval surveillance colonoscopy than recommended by guidelines in small hyperplastic rectal polyps.

**TABLE 5:** Comparison of appropriate responses to clinical vignettes between the gastroenterologists and the colorectal surgeons based on guidelines adherence.

Clinical vignette question	Gastroenterologist *N* (%)	Colorectal Surgeon *N* (%)	*P*-value
Surveillance interval following normal colonoscopy in average-risk patients	48/75 (64.0)	36/112 (32.1)	<0.001
Surveillance interval following normal colonoscopy in high-risk patients	50/74 (67.6)	43/112 (38.4)	<0.001
Surveillance interval following complete endoscopic resection of villous adenoma or tubular adenoma (larger than 1 cm)	40/74 (54.1)	30/112 (26.8)	<0.001
Surveillance interval following resection of hyperplastic polyps in recto-sigmoid region (smaller than 1 cm)	15/75 (20.0)	5/112 (4.5)	0.001

*Data presented as a percentage of appropriate response among responders of each specialty.*

## Discussion

This study revealed that recommendations for CRC screening and awareness of guidelines varied across different groups of physicians. Physicians in the community were less likely to offer screening than specialists practicing in the referral or private hospitals. The main factor determining physicians’ recommendations was the patient’s age. Increasing screening participation rates requires promoting physicians’ recommendations and improving physician awareness and adherence to guidelines.

A population-based study in Thais demonstrated that the screening participation rate increased to 63.0% when the primary care providers were required to offer FOBT for CRC screening in their practice (22). The overall rate of physicians’ recommendations for CRC screening in our study was 58.2%, increasing from a previous report of 32.7% in 2007 (12). However, the distribution of respondents’ area of expertise, workplace, and geography may affect the rate of CRC recommendations in this study. For example, primary care physicians accounted for 13.0% of respondents; therefore, the high rate of screening recommendations may not reflect the practice of the front liners responsible for providing health promotion and screening services. It is worth noting that only about one-third of primary care physicians recommended colon cancer screening even though ∼85.0% of them had access to CRC screening modalities. The study was not designed to explore the reason for this observation, but we hypothesized that the lack of guidelines awareness might be partly responsible for it. Furthermore, the present study showed that 91.0% of colorectal surgeons and 96.0% of gastroenterologists offered screening colonoscopy, a higher than reported number in a previous study (68.0%) (23). Forty-three percent of primary care physicians recommended a colonoscopy, and 60.0% offered FOBT. The data showed that FOBT was the most accessible screening tool for primary care physicians; therefore, it is possible that access to screening tools influenced the physicians’ selection of screening methods.

There is a broad variety of options for CRC screening in the average risk population, including stool tests, such as different types of FOBT, fecal DNA testing, double-contrast barium enema, virtual colonoscopy, and endoscopic procedures. Based on a balance between availability, cost, and potential benefits and safety profile, FOBT is the simplest, most non-invasive, and least expensive screening method. Direct comparison of FOBT showed that people were more likely to pursue an iFOBT than a gFOBT (52.7 vs. 43.9%) because iFOBT was easier to use without diet restriction. The study also showed that iFOBT was more superior to gFOBT in detecting advanced neoplasms. The advanced adenoma detection rate of iFOBT was 1.4% and gFOBT was 0.5%. The iFOBT had a sensitivity of 67.0% and a specificity of 85.0%, whereas gFOBT had a sensitivity of 54.0% and a specificity of 80.0% (24). Therefore, the iFOBT is now recommended as the first-option FOBT for CRC screening. For the present study, both FOBT methods were included because both tests were available and were used in our country when the study was conducted.

A meta-analysis showed that FOBT, flexible sigmoidoscopy, and colonoscopy had no differences in all-cause mortality (25). Studies comparing the efficacy between iFOBT and colonoscopy showed that both methods were comparable in terms of CRC detection, but iFOBT was inferior to colonoscopy in detecting non-advanced adenoma and advanced adenoma. However, iFOBT had higher participation compared to colonoscopy and higher acceptance may counter balance its lower detection ability (26, 27). The cost-effectiveness and budget impact analyses revealed that colonoscopy was more cost-effective in a low-and middle-income country, with an Incremental Cost-Effectiveness Ratio (ICER) of United States Dollars (USD) 646.5/Quality-Adjusted Life Year (QALY) gained (28). However, providing a nationwide screening colonoscopy can be a challenge in Thailand due to low numbers of endoscopists, less than 1,000 for an estimated number of cases requiring CRC screening of 14 million (29). Hence, a population-based one-step screening colonoscopy program is less likely to succeed in resource-limited countries with a shortage of endoscopists. A two-step approach employing FOBT to select average-risk people for colonoscopy is another appropriate CRC screening strategy. The usage of FOBT is encouraging since a screening uptake study revealed that the rural population has easier access to non-invasive screening tests through their primary care physicians (30). However, a population-based study on CRC screening strategy showed that 28.0% of patients with positive FOBT did not undergo colonoscopy (22).

Raising public awareness of CRC and altering people’s attitudes and beliefs is crucial to impact the local community in this major health issue. Participation rates in CRC screening were greater when educational pamphlets were distributed to participants (31–33). Another strategy for increasing CRC screening rates is for clinicians to offer either FOBT or endoscopic procedures if the patient prefers one type of test over the others (34–36). This finding underscores the necessity of providing various screening options whenever available to reach the greatest number of patients.

One pitfall in the choice of the modality of CRC screening was that 10.0–16.0% of primary care physicians, internists, and general surgeons advocated blood CEA level as a screening method. Serum CEA has low sensitivity for diagnosing and screening CRC; therefore, it should not be recommended as a screening tool. Also, serum CEA levels can be elevated in various benign conditions and most types of adenocarcinoma, including breast, gastric, lung, and pancreatic cancers (37–39). This finding is a setback that would hinder Thailand’s efforts to improve CRC screening programs.

Several studies have explored the factors that influence physicians’ recommendations and screening adherence. Patient-related factors such as age, race, and sex have been demonstrated to affect the selection of screening modalities (e.g., colonoscopy vs. FOBT) (40–43). Physicians were less likely to offer screening to patients with chronic conditions, low levels of education, and poor socioeconomic status (40–44). The present study showed that the only patient-related factor influencing the physicians’ decision to offer CRC screening was age. Physicians’ implementation of CRC screening was unaffected by other demographics, family history of CRC, or comorbid illnesses.

The physicians’ adherence to the practice guidelines was also assessed. For interval surveillance colonoscopy following colonoscopy with polypectomy (45, 46), gastroenterologists were more likely to adhere to the guidelines than colorectal surgeons. Both specialties preferred early surveillance colonoscopy for small hyperplastic polyps in the recto-sigmoid region. Shorter interval surveillance colonoscopy may result in unnecessary colonoscopies and increased healthcare costs. This finding reinforces the need to tailor surveillance colonoscopy in order to promote optimal CRC screening utilization.

A strength of our study is that it was a nationwide survey that included all specialties who participate in the national CRC screening program. Also, large samples (586 physicians) responded. The outcomes provided many instances of the drawbacks mentioned earlier or inadequacies, which could be eliminated to help improve the national CRC screening strategy. However, a known limitation of the survey study design is that our survey responses may not reflect the respondents’ actual practices or attitudes. More than half of the respondents worked in the central part of the country, indicating that these practitioners were representative of urban rather than rural area healthcare professionals. Also, the question concerning when to start screening (Item 2.3) can be difficult to answer directly because the information about family history of CRC was not provided. Asymptomatic individuals who have a first-degree relative with CRC diagnosed at a young age would require earlier surveillance. Lastly, the questionnaire did not differentiate iFOBT from gFOBT to determine whether the type of FOBT influenced the decision to offer CRC screening.

In conclusion, recommendations for CRC screening and awareness of the guidelines varied among different specialists. The necessity for CRC screening should be emphasized among primary care physicians. The factors that influence colorectal surgeons’ and gastroenterologists’ lack of adherence to CRC guidelines in small hyperplastic rectal polyps should be explored, and the need for proper interval surveillance colonoscopy should be underscored.

## Data Availability Statement

The original contributions presented in the study are included in the article/Supplementary Material, further inquiries can be directed to the corresponding author.

## Ethics Statement

The study protocol conformed to the Ethical Guidelines of the 1975 Helsinki Declaration, and it was approved by the Siriraj Institutional Review Board (COA no. Si448/2015). The patients/participants provided their written informed consent to participate in this study.

## Author Contributions

NP: conception and supervision. NP and PC: methodology and writing – review and editing. NP, PT, TG, and PC: formal analysis. PT: data curation. PT and TG: writing – original draft preparation. All authors contributed to the article and approved the submitted version.

## Conflict of Interest

The authors declare that the research was conducted in the absence of any commercial or financial relationships that could be construed as a potential conflict of interest.

## Publisher’s Note

All claims expressed in this article are solely those of the authors and do not necessarily represent those of their affiliated organizations, or those of the publisher, the editors and the reviewers. Any product that may be evaluated in this article, or claim that may be made by its manufacturer, is not guaranteed or endorsed by the publisher.

## Footnotes

1
www.surveymonkey.com
