# Supplementary material for: An Assessment of Physicians’ Recommendations for Colorectal Cancer Screening and International Guidelines Awareness and Adherence: Results From a Thai National Survey
Source: Front Med (Lausanne). 2022 Apr 29;9:847361. doi: 10.3389/fmed.2022.847361 (PMC9100397; doi:10.3389/fmed.2022.847361)
Supplement: Supplementary file 1 [file Data_Sheet_1.docx]

**Questionnaire**

**Part 1: Demographic data**

- 1. Age

…………… years

- 1. Gender

🞏 Male 🞏 Female

- 1. What is your specialty?

🞏 Primary care physician 🞏 Internist

🞏 Gastroenterologist 🞏 General surgeon

🞏 Colorectal surgeon 🞏 Resident physician in surgery

🞏 Resident physician in internal medicine

- 1. How many years have you been practicing after graduation?

🞏 < 5 years 🞏 5-10 years 🞏 11-15 years

🞏 16-20 years 🞏 > 20 years

- 1. How many patients do you see per week?

🞏 < 20 patients/week 🞏 21-49 patients/week

🞏 50-100 patients/week 🞏 >100 patients/week

- 1. What type of hospital do you work at?

🞏 Community hospital 🞏 Provincial hospital

🞏 Tertiary care center 🞏 Academic center

🞏 Private clinic 🞏 Private hospital

- 1. Where is your hospital located?

🞏 Central region 🞏 Northern region 🞏 Southern region

🞏 Eastern region 🞏 Northeastern region 🞏 Western region

**Part 2: Practice pattern in Colorectal cancer (CRC) screening**

2.1 Have you seen any consults for CRC screening in the past one year?

🞏 Yes 🞏 No

2.2 Have you recommended your patients for CRC screening in the past one year?

🞏 Yes 🞏 No

2.3 At what age do you start recommending asymptomatic patients for CRC screening?

🞏 45 years 🞏 50 years 🞏 55 years 🞏 60 years 🞏 Not recommend

2.4 Which of the followings affect your decision to recommend CRC screening?

(Can choose more than one answer)

🞏 Age 🞏 Gender 🞏 Family history of CRC

🞏 Comorbidity 🞏 Hospital facility 🞏 Reimbursement policies

2.5 What are the available modalities for CRC screening at your hospital?

(Can choose more than one answer)

🞏 FOBT 🞏 Barium enema 🞏 Sigmoidoscopy

🞏 Colonoscopy 🞏 CT colonography 🞏 CEA

2.6 If reimbursement is available, would you offer your patients CRC screening?

🞏 Yes 🞏 No

🞏 Reimbursement does not affect my decision

2.7 In the past year, have you recommended CRC screening to your patients with a family history of colon cancer?

🞏 Yes 🞏 No

🞏 I have not encountered patients with a family history of colon cancer last year.

2.8 Which screening modalities do you offer to your patients?

🞏 FOBT 🞏 Sigmoidoscopy 🞏 Barium enema + sigmoidoscopy

🞏 Colonoscopy 🞏 CT colonography 🞏 CEA

2.9 At what age do you stop recommending CRC screening or surveillance?

🞏 65 years 🞏 70 years 🞏 75 years 🞏 80 years

2.10 Are you aware of international guidelines for CRC screening?

🞏 Yes 🞏 No 🞏 Not sure

***If you are a gastroenterologist or surgeon, please answer questions 3.1-3.4.***

If you are not a gastroenterologist or surgeon, you are not required to answer questions 3.1-3.4.

**Part 3: Adherence to CRC screening guidelines**

3.1 When do you recommend surveillance in a patient without a family history of CRC who had normal screening colonoscopy?

🞏 10 years 🞏 5 years 🞏 3 years 🞏 1 year 🞏 No surveillance

3.2 When do you recommend surveillance in a patient with a family history of CRC who had normal screening colonoscopy?

🞏 10 years 🞏 5 years 🞏 3 years 🞏 1 year 🞏 No surveillance

3.3 When do you recommend CRC surveillance for a patient who had complete polypectomy of villous or tubulovillous adenoma of larger than 1 cm?

🞏 10 years 🞏 5 years 🞏 3 years 🞏 2 years 🞏 1 year

3.4 When do you recommend surveillance for a patient who had multiple (<10 polyps) hyperplastic polyps of less than 1 cm at the sigmoid colon and rectum?

🞏 10 years 🞏 5 years 🞏 3 years 🞏 2 years 🞏 1 year
